# Assessment of Conventional and Low Gossypol Cottonseed Meal as Alternative Protein Sources in Low-Fishmeal Diets of Hybrid Grouper (*Epinephelus fuscoguttatus* *♀* *× Epinephelus lanceolatus* *♂*): Growth, Feed Utilization, Gut Histology, and Immunity

**DOI:** 10.3390/ani12151906

**Published:** 2022-07-26

**Authors:** Misbah Irm, Bo Ye, Xiaoyi Wu, Lina Geng, Qinxiao Cai, Lu Zhang, Haoyun Zhai, Zhiyu Zhou

**Affiliations:** 1 State Key Laboratory of Marine Resource Utilization in South China Sea, Hainan University, Haikou 570228, China; misbahirm@yahoo.com (M.I.); yeb5@haid.com.cn (B.Y.); 21110710000010@hainanu.edu.cn (L.G.); cqx942437703@163.com (Q.C.); zl312169@163.com (L.Z.); z17666054675@163.com (H.Z.); hndxzhouzy@163.com (Z.Z.); 2 Hainan Provincial Key Laboratory for Tropical Hydrobiology and Biotechnology, Department of Aquaculture, Hainan University, Haikou 570228, China

**Keywords:** hybrid grouper, cottonseed meal, growth performance, feed utilization

## Abstract

**Simple Summary:**

The rapid and sustained growth rate of global aquaculture has forced the aquaculture industry to explore alternative and more sustainable feed ingredients. Plant protein ingredients are promising substitutions for fish meal in the aquaculture industry. The current study aimed to investigate the potential influence of replacing poultry by-product meal protein with conventional cottonseed meal protein (CCMP) or low-gossypol cottonseed meal protein (LGCMP) on growth, feed utilization, gut micromorphology, and immunity of hybrid grouper (*Epinephelus fuscoguttatus* *♀* *× Epinephelus lanceolatus* *♂*) juveniles fed low fish-meal (18.53%, dry matter) diets. Results demonstrated that, without any reduction in fish performance, 80% dietary PBMP could be replaced by CCMP, while this replacement level was 40% for LGCMP. Survival remained unaffected in all dietary treatments, and no fish mortality was observed during the growth trial. These results suggest that cottonseed meal could be a suitable alternative protein source for hybrid grouper farming.

**Abstract:**

A 9-week growth trial was carried out to assess the influence of replacing poultry by-product meal protein with conventional cottonseed meal protein (CCMP) or low gossypol cottonseed meal protein (LGCMP) on growth, feed utilization, gut micromorphology, and immunity of hybrid grouper (*Epinephelus fuscoguttatus* *♀* *× Epinephelus lanceolatus* *♂*) juveniles fed low-fish meal (18.53%, dry matter) diets. Eleven experimental diets were prepared. The control diet (PBMP) contained 46.15% poultry by-product meal protein. Both conventional cottonseed meal protein (CCMP) and low-gossypol cottonseed meal protein (LGCMP) were used in replacement ratios of 20, 40, 60, 80, and 100% of poultry by-product meal protein (PBMP) from the control diet, forming ten experimental diets (CCMP20, CCMP40, CCMP60, CCMP80, CCMP100, LGCMP20, LGCMP40, LGCMP60, LGCMP80, and LGCMP100). Results demonstrated that weight-gain percentage (WG%) was not different between different sources of cottonseed meal (CCMP and LGCMP). However, values of WG% significantly differed among different replacement levels, with CCMP80 and LGCMP40 having significantly higher values compared to other treatments. Fish fed CCMP80 and LGCMP40 exhibited higher protein efficiency ratios (PERs) than fish fed other experimental diets. The regression analysis from a second-order or third-order polynomial model based on WG% showed that the optimal PBMP replacement levels by CCMP and LGCMP are 74% and 33%, respectively. The whole-body lipid contents remarkably decreased as dietary CCMP or LGCMP inclusion levels increased. The relative mRNA expression of insulin-like growth factor-1(IGF-1) in liver was higher in fish fed CCMP80 and LGCMP40 diets compared to fish fed other diets. Generally, in low-FM diets of hybrid grouper, CCMP and LGCMP could replace 74% and 33% of PBMP, respectively.

## 1. Introduction

Fish meal (FM) is an important source of energy, protein, essential amino acids, and fatty acids in aquatic feeds [1]. However, the gradually increasing cost of FM and expansion in aquaculture industry has made fish meal feed a limiting factor in the aquaculture sector [2,3]. Therefore, researchers and nutritionists are focusing on low-FM diets. To date, several alternative protein sources, including animals and plant protein sources, have been tested to reduce the use of FM in aquaculture industry.

Poultry by-product meal (PBM) is an important alternative protein source of FM, especially for carnivorous fish, due to higher protein contents, palatability, and favorable amino acid profile [4,5]. Our previous study demonstrated that fish meal could be replaced with PBM to up to 70% in the diet of juvenile hybrid grouper without impairing growth performance [6]. However, higher replacement (50–70%) in our previous research resulted in steatosis in hepatocytes and higher lipid deposition in abdominal cavity [6]. Steatosis in hepatocytes and higher lipid deposition in abdominal cavity might be due to higher lipid contents and the deficiency of eicosapentaenoic and docosahexaenoic acid of the diets supplemented with high levels of PBM. Therefore, it is important to reformulate and substitute PBM protein with other suitable alternative protein sources to culture healthy fish.

Cottonseed meal (CSM) is an appropriate plant protein source and is cheaper than FM and other protein sources due to the world-wide production of cotton. CSM protein has been used as an attractive substitute of FM, and it is considered as a quality protein ingredient for fish due to its higher protein content (60%) and adequate availability [7]. A major limitation to the use of CSM in animal feeds is gossypol. Gossypol has an anti-nutritional influence on fish and warm-blooded animals fed cottonseed products [8]. However, various processing methods have been applied to eliminate anti-nutritional factors and gossypols from oilseed by-products including cottonseed meal, soybean meal, and rapeseed meal for the potential use of these protein sources in animal feed [9].

The potential of cottonseed meal as a replacer of fish meal and soybean meal has been tested in several fish such as juvenile southern flounder *Paralichthys lethostigma* [10], juvenile black sea bass *Centropristis striata* [11], hybrid striped bass *Morone saxatilis* *♀* *× Morone chrysops* *♂* [12], Florida pompano *Trachinotus carolinus* [13], Nile tilapia, *Oreochromis niloticus*, and juvenile common carp *Cyprinus carpio* [14,15] without impairing their growth. It is well-known that nutritional factors, including amino acid imbalance and dietary protein, affect the regulation of insulin-like growth factor-1 [16]. The main environmental factor regulating the GH-IGF system is the nutritional status [17]. Many studies conducted on alternative protein sources, including animal and plant protein sources, have indicated that hepatic IGF-1 expression of fish was significantly influenced by the inclusion of these sources [18,19,20].

Inclusions of cottonseed meal displayed differential results regarding intestinal morphology in different fish species. Fish intestine is considered as the main target in nutritional challenges, as it plays a key role in the digestion, absorption of food, and metabolism of dietary nutrients, as well as in immunity [21]. The intestinal status in response to nutritional changes has been extensively assessed in different fish species. Particularly, influence of low fish-meal diets on the gut physiology of different species has been evaluated in different stages of growth [22,23]. Several studies suggested that high incorporation of plant protein ingredients in the fish diet affected the gut health and resulted in morphological alterations in intestine due to anti-nutrients [24,25]. For example, incorporation of cottonseed meal in the diet of asallogynogenetic silver crucian carp *Carassius auratus gibelio* *♀* *× Cyprinus carpio* *♂* did not affect gut morphology [26], while CSM incorporation in the diet of juvenile turbot *Scophthalmus maximus* L. reduced villi height, microvilli height [24].

Various studies showed that the inclusion of cottonseed meal in the diet of different fish species improved immune indices, including LZM and IgM concentrations in serum and intestine [27,28,29]. Humoral components such as immunoglobulins and lysozyme played an important role in non-specific and specific immunity of fish [30]. Non-specific immune system is crucial for disease resistance and indicates health status of fish [31], and it depends on sufficient nutrients supplied by the feed. Therefore, dietary status is the key factor which affects immunity in fish [32]. LZM and IgM concentrations were used to assess the health of aquatic organisms [33]. Lysozyme was considered as a vital component of non-specific immune system, as it had antibacterial activity and cleaved bacteria by damaging cell walls [34]. IgM was one of the most important immunoglobulins in fish humoral adaptive immunity [35].

Hybrid grouper *(Epinephelus fuscoguttatus* *♀* *× Epinephelus lanceolatus* *♂)* is a new marine fish in Asian countries, exhibiting better growth performance compared with the parental fish [36]. This fish species is gaining much attention in mariculture due to its rapid growth and high disease resistance [37], as well as its strong adaptability to salinity [38]. Dietary protein and lipid requirements of juvenile hybrid grouper have been established in previous studies [39,40]. Limited studies on the replacement limits of alternative protein sources in the diet of hybrid grouper are available [27,41].

The objective of this study was to evaluate the influence of replacing poultry by-product meal protein with conventional cottonseed meal protein or low-gossypol cottonseed meal protein on growth, feed utilization, gut micromorphology, and immunity of hybrid grouper juveniles fed low fish-meal diets.

## 2. Materials and Methods

### 2.1. Ethics

We declare that this study was conducted following both “3R” (Replacement, Reduction, Refinement) rules and Hainan University Application for Animal Welfare and Ethical Review (HNUAUCC-2021-00032).

### 2.2. Experimental Diets

Eleven experimental diets were prepared to replace 0, 20, 40, 60, 80, and 100% of poultry by-product meal protein (PBMP) by conventional cottonseed meal protein (CCMP) or low-gossypol cottonseed meal protein (LGCMP) at a low dietary fish-meal level (18.53%, dry matter), being designated as PBMP, CCMP20, CCMP40, CCMP60, CCMP80, CCMP100, LGCMP20, LGCMP40, LGCMP60, LGCMP80, and LGCMP100, respectively, as shown in Table 1. The control diet contained 46.15% of poultry by-product meal. All experimental diets were closely isoproteic (45.83 ± 0.413% dry matter) and isolipidic (8.4 ± 0.10% dry matter). Crystalline essential amino acids were individually added to balance the amino acid profile of all dietary treatments based on previous study [42]. Mineral and vitamin premix were added according to those described in our previous study [40]. Carboxymethyl cellulose (CMC) was added as a binder. The preparation of experimental diets was carried out by following the same procedure as previously described by Zhou et al. [43]. Table 2 displays the analytical amino acid profile of the experimental diets.

### 2.3. Growth Trial and Zootechnic Performance

Experimental fish were bought from a commercial hatchery (Lingao, China). Fish were acclimated with commercial feed for 2 weeks before the onset of growth trial. After the acclimation, experimental fish were randomly allocated into 33 tanks (L 120 cm × W 70 cm × H 50 cm, three tanks per dietary group) at a stocking density of 14 fish per tank (average initial body weight: 5.27 ± 0.05 g/fish). These tanks were connected to a recirculating system, and sea water (salinity: 33.1 g/L) was provided to all tanks from the same reservoir. Fish were fed test diets to apparent satiation twice a day for 9 weeks. Water quality was monitored every day. Water temperature ranged from 27 to 29 °C; dissolved oxygen ranged from 5.8 to 6.8 mg/L, and total ammonia ranged from 0.to 0.20 mg/L. Fish were subjected to a 12 h light–12 h dark cycle. Fish zootechnic performances such as survival, weight gain, and feed conversion were measured weekly.

### 2.4. Sampling and Analysis

At the start of the experiment, 10 fish were sampled to determine the initial chemical composition of whole body. At the termination of the growth trial, fish were starved for 14 h before sampling.

#### 2.4.1. Whole Body, White Muscle Composition, and Somatic Indices

After being anesthetized with MS-222 (0.1 g/L), two fish per tank were sampled to determine the proximate composition of whole body. Three fish from each tank were weighed and dissected to collect visceral tissues after the blood collection from caudal vein to determine the somatic indices. White muscle was taken off at this dissection. At this dissection, liver and gut samples were also obtained for further analysis. The moisture contents in the whole body, white muscles, and diets were determined after drying samples in an oven at 105 °C until constant weight. Crude protein (N × 6.25) contents were analyzed via Dumas combustion method using a rapid MAX N exceed system (Elementar, Langenselbold, Germany). Crude lipid was determined by the ether extraction method (Soxtec System HT6, Haineng SOX406, Jinan, China).

#### 2.4.2. Serum IgM and LZM Concentrations

Serum IgM concentrations and lysozyme activity (LZM) were measured with ELISA kits (Kit no: C0197170176, F18019756; Cusabio, Wuhan, China) following the procedure suggested by manufacturer.

#### 2.4.3. Histological Analyses

For gut histological examination, saline solution was used to wash the gut samples, and then samples were fixed in Davidson’s fixative solution for 24 h. After dehydrating with ethanol, the samples were fixed in paraffin wax and transversely cut into sections of 4 μm. The sections were stained by hematoxylin and eosin and then examined using light microscope (Olympus IX71) and Image-Pro Plus 7.0 software. Digitalized images were analyzed to measure the micrometer length of various enteric structures. Average fold height and microvillus height (hMV) were determined per slice (10 folds per individual sample) according to the procedures described by Escaffre et al. [44].

#### 2.4.4. Molecular Analysis (Real-Time Quantitative PCR Analysis of IGF-1)

Total RNA of the samples (liver) was isolated using Trizol Reagent (Invitrogen, Waltham, MA, USA). The integrity of RNA was examined with 1% agarose gel. The nucleotide sequence (F: TATTTCAGTAAACCAACAGGCTATG R: TGAATGACTATGTCCAGGTAAAGG) of targeted gene (*IGF-1*) was based on previous published nucleotide sequence [43]. Real-time quantitative PCR analysis was performed in quantitative thermal cycler (QuantStudio 6 Flex, Applied Biosystems, Waltham, MA, USA) using SYBR Green Premix Ex Taq TMП (Takara, Japan). The qRT–PCR amplification conditions were as follows: 95 °C for 5 s, 56 °C for 30 s, and 72 °C for 30 s, followed by 95 °C for 15 s, 60 °C for 1 min, and 95 °C for 15 s. After qRT–PCR, melting curve analysis of amplification products was performed to confirm the presence of a single PCR product in these reactions. Five different dilutions (in triplicate) of complementary DNA samples were used to create standard curves, and amplification efficiency was calculated using the equation: E = 10^(−1/slope)^ − 1. The expression of the target genes were analyzed following 2^−ΔΔct^ method [45].

### 2.5. Calculations

The parameters were calculated as follows:


Weight gain (WG%) = 100 × (final average body weight − initial average body weight)/initial average body weight
(1)

Feed conversion ratio (FCR) = total dry feed fed (g)/total body weight gain (g)
(2)

Daily feed intake (DFI) = 100 × dry feed intake (g)/weight gain (g)/number of days
(3)

Protein efficiency ratio (PER) = weight gain (g)/protein intake (g)
(4)

Survival% = 100 × (finial number of fish)/(initial number of fish)
(5)

Condition factor (CF) = 100 × body weight (g)/body length (cm)^3^ (6)

Hepatosomatic index (HSI) = 100 × liver weight (g)/whole-body weight (g)
(7)

Intraperitoneal fat (IPF) = 100 × intraperitoneal fat weight (g)/body weight (g)
(8)

### 2.6. Statistical Analysis

Normality and homoscedasticity of all data were checked before the statistical analysis. The independent and interactive effects of cottonseed meal sources and replacement levels were analyzed using two-way ANOVA. Tukey’s multiple range test was used to determine the differences among means at 5% probability level (*p* < 0.05). All data were processed with SPSS 20.0 (IBM, Armonk, NY, USA). The regression analysis from a second-order or third-order polynomial model was performed to assess the responses to graded replacing levels of PBMP with CCMP or LGCMP.

## 3. Results

### 3.1. Survival and Zootechnic Performances

**Figure 1:**
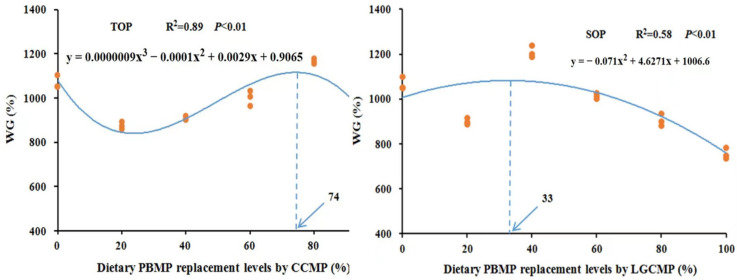
Relationship of WG% of hybrid grouper juveniles with different dietary PBMP replacement levels by CCMP or LGCMP.

Results for survival, growth performance, and feed utilization of hybrid grouper are presented in Table 3. There was no significant difference in survival, and no fish mortality was observed in any group during the growth period. Two-way ANOVA analysis showed that substitution of PBMP with CCMP or LGCMP had no significant influence on WG%. However, different PBMP replacement levels by CCMP or LGCMP showed a significant effect on WG% (*p* < 0.05). Fish fed CCMP80 and LGCMP40 exhibited significantly higher values of WG% as compared to fish fed with other dietary groups. The regression analysis from a second-order or third-order polynomial model based on WG% showed that the optimal PBMP replacing levels by CCMP and LGCMP for the maximal growth of hybrid grouper were estimated to be 74 and 33%, respectively; see Figure 1.

Feed utilization parameters (PER, FCR, and DFI) exhibited significant difference between CSM sources and among different PBM replacement levels (*p* < 0.05). Fish fed CCM source showed significantly higher PER values than fish fed LGCM source. Among different PBMP replacement levels, fish fed CCMP80 and LGMP40 demonstrated higher PER values than fish fed other diets. FCR and DFI values were found to be higher in fish fed LGCM source than those in fish fed CCM source. While in different PBMP replacement levels, fish fed CCMP100 and LGCMP100 displayed higher FCR and DFI than fish fed other diets (*p* < 0.05). There was significant interaction in WG%, PER, FCR, and DFI between CSM sources and PBMP replacement levels (*p* < 0.05).

### 3.2. Somatic Indices

There was no significant difference in somatic indices (HSI and CF) between CSM sources or among all dietary replacement levels; see Table 4. While fish fed with CCM source displayed higher IPF values than fish fed with LGCM source, there was no significant difference among different PBMP replacement levels with CCMP or LGCMP. No significant interaction was found between the CSM sources and the PBMP replacement levels for CF, HSI, and IPF.

### 3.3. Whole-Body and White Muscle Compositions

Whole-body and white muscle compositions of all feeding groups are shown in Table 5. Whole-body moisture contents did not show significant difference between CSM sources. However, among all replacement levels, the treatment of 100% dietary PBMP replacement had the highest moisture contents. There was no significant interaction between CSM sources and the replacement levels for moisture contents in whole body. The whole-body protein contents were significantly higher in fish fed LGCM source in comparison to those fish fed CCM source, but they were not significantly affected by different PBMP replacement levels. No significant interaction was found between the CSM sources and the PBMP replacement levels in whole-body protein contents.

Whole-body lipid contents were remarkably decreased as the PBMP replacement levels increased (*p* < 0.05). Fish fed the diets CCMP100 and LGCMP100 exhibited lower whole-body lipid contents than fish fed other diets (*p* < 0.05). There was no significant interaction between the CSM types and the PBMP replacement levels in whole-body lipid contents.

In white muscle compositions, moisture contents were significantly different between CSM sources (*p* < 0.05), and higher moisture contents were found in fish fed CCM source compared to those in fish fed LGCM source. Among different PMBP replacement levels, fish fed at 100% dietary PMBP replacement exhibited higher moisture contents (*p <* 0.05) than fish fed at other PMBP replacement levels. No significant interaction was observed in white muscle moisture contents between the CSM types and the PBMP replacement levels.

White muscle protein contents differed significantly between CSM sources as well as among different replacement levels. Fish fed the LGCM source displayed higher protein contents than fish fed the CCM source (*p* < 0.05). In PBMP replacement levels, fish fed the LGCMP40 diet had higher white muscle protein contents than those fed other diets (*p* < 0.05). There were significant interactive effects on white muscle protein contents between the CSM sources and the PBMP replacement levels (*p* < 0.05). Lipid contents in white muscles were not significantly different between CSM sources. However, treatments of 20 and 40% PBMP replacement levels had higher lipid contents than other replacement levels. No significant interaction was noticed between the CSM sources and the PBMP replacement levels for lipid contents in white muscles.

### 3.4. Gut Histology

**Figure 2:**
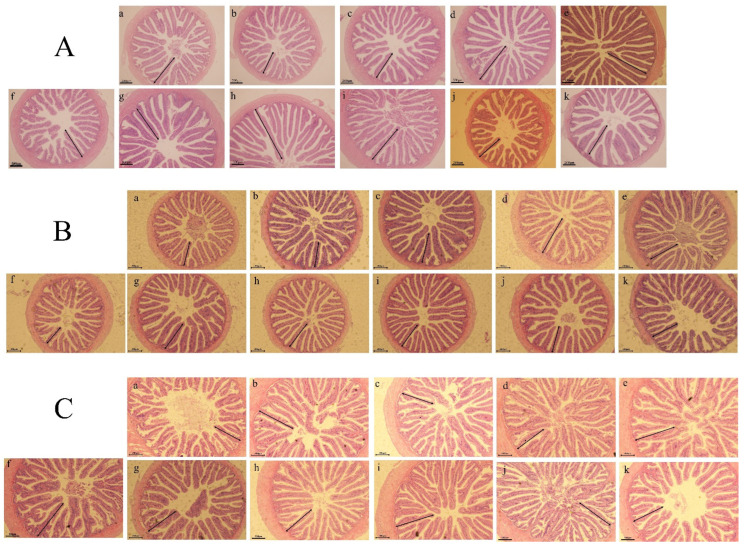
Light microscopy of gut morphology of hybrid grouper juveniles in fish fed different PMBP replacements by CCMP and LGCMP for 9 weeks (hematoxylin–eosin staining; original magnification 10×), (**A**): foregut, (**B**): midgut, (**C**): hindgut; (a): PBMP, (b): CCMP20, (c): CCMP40, (d): CCMP60, (e): CCMP80, (f): CCMP100, (g): LGCMP20, (h): LGCMP40, (i): LGCMP60, (j): LGCM80, (k): LGCMP100.

Results of gut histology are shown in Table 6 and histological examination images are shown in Figure 2. Fold height and Microvillus height (hMV) values in fore gut were significantly different among all replacement levels. These values were higher in fish fed CCMP80 and LGCMP40 diets than those fed other diets, and hF did not display significant variations between CSM sources in foregut. However, there was a significant difference in hMV in the foregut between the CSM sources, with greater hMV values in fish fed the LGCM source compared to fish fed the CCM source (*p* < 0.05).

In midgut and hindgut, hF displayed considerable variations between both CSM sources and among all replacement levels (*p* < 0.05), while hF values were higher in fish fed the CCM source than those fed the LGCM. Among PBMP replacement levels, fish fed the diets CCMP60, CCMP80, and LGCMP40 had higher hF compared to fish fed the other diets, while hMV in midgut and hindgut exhibited considerable differences among all replacement levels. Fish fed dietary treatments (LGCM40 and CCMP80) had higher hMV values compared to fish fed other dietary groups (*p* < 0.05). Further, hMV in the midgut did not reveal clear differences between CSM sources. However, in the hindgut, hMV showed significant differences between CSM sources, with higher values in fish fed CCMP compared to fish fed LGCMP (*p* < 0.05).

### 3.5. Expression of Hepatic IGF-1

**Figure 3:**
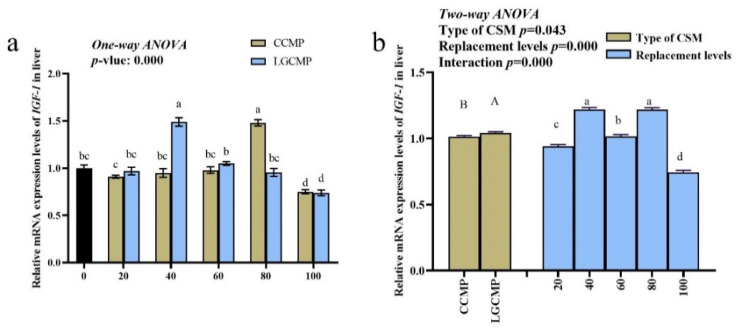
Expression of hepatic *IGF-1* in hybrid grouper juveniles fed low-FM diets with different PBMP replacements by CCMP or LGCMP for 9 weeks. The gene expression of the PBMP group was set as 1. Figure (**a**) represented one-way ANOVA results and figure (**b**) represented Two-way ANOVA results. Subscript letters a,b,c,d showed significant differences among different PBMP replacement levels by CCMP or LGCMP and subscript letters A,B represented significant difference between CSM sources.

The mRNA levels of hepatic IGF-1 exhibited considerable variations between the CSM sources and among different PBMP replacement levels (Figure 3). The hepatic IGF-1 expression was higher in fish fed LGCCM source compared to fish fed CCM source. Among different PBMP replacement levels, mRNA levels of hepatic IGF-1 significantly upregulated in fish fed CCMP80 and LGCMP40 over those fed other diets. There was a significant interaction between the CSM types and the PBMP replacement levels for expression of *IGF-1* (*p* < 0.05).

### 3.6. Serum IgM and LZM Concentrations

**Figure 4:**
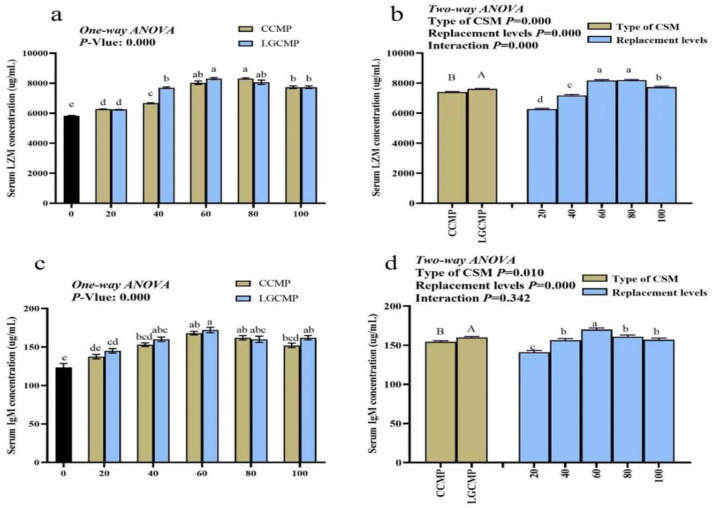
The concentration of LZM and IgM activity of in serum of hybrid grouper juveniles fed low-FM diets with different PMBP replacements by CCMP or LGCMP for 9 weeks. Superscript letters (a,b,c,d) represented significant difference among different PBMP replacement levels by CCMP or LGCMP while superscript letter (A,B) represented significant difference between CSM sources. Subfigures (**a**,**b**) explains One way ANOVA and Two-way ANOVA results for LZM concentration and subfigures (**c**,**d**) explains one way ANOVA and Two-way ANOVA results for IgM concentration.

Serum LZM concentrations showed significant differences between the CSM sources, with higher LZM concentrations in fish fed LGCM source compared to fish fed the CCM source (Figure 4). Among PBMP replacement levels, fish fed the diets PBMP, CCMP20, CCMP40, and LGCMP20 showed significantly lower serum LZM concentrations than fish fed CCMP60, CCMP80, CCMP100, LGCMP40, LGCMP60, LGCMP80, and LGCMP100. There was significant interaction between CSM types and the PBMP replacement levels for serum LZM concentrations (*p* < 0.05).

Serum IgM concentrations differed significantly between the CSM sources and among different replacement levels (*p* < 0.05). Serum IgM concentration was higher in fish fed LGCM source. In different PBMP replacement levels, fish fed the dietary treatments PBMP, CCMP20, LGCMP20 displayed significantly lower IgM concentrations compared with fish fed other diets. There was no significant interaction between the CSM types and the PBMP replacement levels for serum IgM concentrations (Figure 3). Superscript letters (a,b,c,d) represented significant difference among different PBMP replacement levels by CCMP or LGCMP While superscript letter (A,B) represented significant difference between CSM sources.

## 4. Discussion

### 4.1. Zootechnic Performances

Cottonseed meal (CM) protein has been included in the feed of several fish species in different forms to replace FM without impairing their growth, such as low-gossypol cottonseed meal [10,11,25], fermented cottonseed meal [46], cottonseed flour [47], and regular cottonseed meal [48]. However, dietary FM levels in some studies were still high: 46% in silver sillago [25], 48% in juvenile black sea bream [46], 41% in parrot fish [48] and 27.5–45.6% in pearl gentian grouper [49,50]. In the present study, the low-FM (18.53%) basal diet achieved similar growth as the whole-FM diet in our previous research [6]. Results demonstrated that fish fed both sources of CSM did not exhibit significant difference in growth performance and feed utilization parameters. Similar results were reported in juvenile southern flounder when fed with different CSM sources [10]. However, different replacement levels showed significant differences in terms of weight-gain percentage (WG%) and protein efficiency ratio (PER). Similarly, 80% dietary PBMP could be replaced by CCMP, while the replacement level was 40% for LGCMP without showing any adverse effect on fish performance. These findings suggest that CCM is a quality protein ingredient in the diet of hybrid grouper when combined with animal protein sources such as PBMP and that combination of animal and plant protein sources such as cottonseed meal and poultry meal might be the better choice to formulate low fish-meal diets in aquafeeds. Alam et al. [10] also reported that CSM is an appropriate protein ingredient when combined with other protein sources such as soybean meal or poultry meal in the diet of juvenile southern flounder.

Biochemical, physiological, and molecular mechanisms could be determined through the interaction between nutrition and gene function concerning the changes in feed formulation [51]. The relative mRNA level of *IGF-I* gene in fish tissues is measured as a growth indicator [52]. IGFs regulate many biological functions, including cell division, cell proliferation, and cell growth. In the present study, both sources and different replacements of PBMP with CSMP in hybrid grouper diets significantly affected the relative expression of *IGF-1*, which showed a strong correlation with growth performance. These findings are consistent with several previous studies [19,24,53].

### 4.2. Whole-Body and White Muscle Compositions

Whole-body and white muscle compositions did not exhibit significant interaction between CSM sources and replacement levels. However, whole-body lipid values decreased with increasing levels of both types of CSM. Previous studies reported differential results about lipid contents in different fish species when fed with CSM. For instance, higher incorporation of CSM reduced the lipid contents in whole body of black sea bass *Centropristis striata* [11] and juvenile red drum *Sciaenops ocellatus* [47]. The lower lipid level in the fish fed higher CSM was probably due to higher fat catabolism with less fat storage. In contrast, Alam et al. [10] reported higher lipid contents in whole body of juvenile flounder *Paralichthys lethostigma* when offered higher CSM diets and suggested that high lipid contents could be due to impaired liver function [10].

### 4.3. Gut Micromorphology

Fish growth is linked with gut health, which is linked to the absorption and digestion of nutrients. Dietary ingredients might affect intestinal morphology [54]. Fish performance, such as in terms of survival and growth, could be compromised by physiological alterations in intestine. Villus height and crypt depth reflect the functional integrity and morphology of the intestine [55]. It is well-known that plant protein sources have anti-nutritional factors which might damage or alter the gut morphology. Therefore, gut micromorphology should be examined precisely while including alternative plant protein sources in fish diets.

In this study, CSM sources and PBMP substitutions with CSM proteins in the diet of hybrid grouper changed the gut morphology, and the dietary groups which resulted in better growth performance also showed better gut micromorphology, disagreeing with the reports of Cai et al. [26], which showed high inclusions of CSM in the diet of asallogynogenetic silver crucian carp *Carassius auratus gibelio* *♀* *× Cyprinus carpio* *♂* did not affect gut morphology. These differential results depend on different fish species and their tolerance to anti-nutrients. This study suggested that hybrid grouper seems to tolerate high levels of plant protein sources. Gut histological evaluation in this study indicated that health aspects agreed with growth performance results of hybrid grouper.

### 4.4. Serum LZM and IgM Concentrations

Non-specific immunity plays an important role for health status and disease resistance of fish [31], and dietary status is the key factor that affects immunity in fish [32]. Lysozyme is considered a vital component of the non-specific immune system as it has antibacterial, antiviral, and anti-inflammatory properties [34,56]. Lysozyme is affected by several external factors including nutritional factors; therefore, it is widely employed in fish nutrition research to assess the health of fish. IgM is also an essential immunoglobulin in fish adaptive immunity and plays important roles in activation of complement and phagocytosis [35,57].

In the current study, fish fed with CCMP20 and LGCMP20 showed low LYZ activity and IgM concentration, indicating that increasing PBMP substitutions with CSM proteins in the diets perhaps enhanced the immunity of hybrid grouper. The increment in LZM and IgM in serum could be attributed to the presence of anti-nutrient factor (gossypol) in CSM, which exhibits antibacterial, antiviral, and anti-parasitic properties, agreeing with the studies of Ye et al. and Yilidrim et al. [27,29]. The various studies revealed varying impacts of dietary modification on the immune response of different fish [58]. For example, higher inclusions of plant proteins in the diet of rainbow trout suppressed the nonspecific immune response [59], while a study performed on Atlantic salmon [60] reported that higher inclusions of plant proteins increased nonspecific immune response.

## 5. Conclusions

This study concluded that, in low-FM diets of hybrid grouper, PBMP could be replaced with CCMP and LGCMP to up to 74% or 33% without reducing growth and feed utilization. Total replacement of PBMP with CCMP and LGMP had a negative influence on fish performance in terms of WG% and PER.

## Figures and Tables

**Table 1:** Formulations (dry matter basis, %) and analyzed compositions of experimental diets.

Ingredients	Dietary Treatments										
	PBMP	CCMP 20	CCMP 40	CCMP 60	CCMP 80	CCMP 100	LGCMP 20	LGCMP 40	LGCMP 60	LGCMP 80	LGCMP 100
Fish-meal (Anchovy) ^1^	18.53	18.53	18.53	18.53	18.53	18.53	18.53	18.53	18.53	18.53	18.53
Poultry by-product meal ^2^	46.15	36.92	27.69	18.46	92.3	0.00	36.92	27.69	18.46	9.23	0.00
Conventional cottonseed proteome ^3^	0.00	9.68	19.37	29.05	38.74	48.42	0.00	0.00	0.00	0.00	0.00
Low-gossypol cottonseed proteome ^4^	0.00	0.00	0.00	0.00	0.00	0.00	9.56	19.11	28.67	38.23	47.78
Chile fish oil (Salmon)	0.00	1.17	2.34	3.50	4.67	5.84	1.17	2.34	3.50	4.67	5.83
Vitamin premix	1.	1.	1	1	1	1	1	1	1	1	1
Mineral premix	0.5	0.5	0.5	0.5	0.5	0.5	0.5	0.5	0.5	0.5	0.5
Corn starch	19.24	19.24	19.24	19.24	19.24	19.24	19.24	19.24	19.24	19.24	19.24
L-Alanine	0.03	0.28	0.28	0.21	0.11	0.01	0.30	0.33	0.25	0.17	0.08
L-Arginine	0.24	0.00	0.00	0.00	0.00	0.00	0.00	0.00	0.00	0.00	0.00
L-Phenylalanine	0.24	0.13	0.03	0.00	0.00	0.00	0.13	0.03	0.00	0.00	0.00
L-Methionine	0.55	0.60	0.65	0.69	0.74	0.79	0.60	0.65	0.72	0.77	0.82
L-Isoleucine	0.12	0.17	0.23	0.28	0.34	0.39	0.15	0.18	0.21	0.24	0.27
Carboxymethyl cellulose sodium	2.00	2.0	2.0	2.0	2.0	2.0	2.0	2.0	2.0	2.0	2.0
Cellulose	11.40	9.77	8.15	6.53	4.91	3.29	9.90	8.41	6.92	5.43	3.93
Proximate compositions											
Dry matter	92.68	91.89	91.99	92.06	92.47	92.91	93.07	92.88	92.55	92.49	91.86
Crude protein (%, dry matter)	45.85	45.68	46.38	46.19	45.59	45.66	44.99	45.43	46.11	46.28	45.97
Crude lipid (%, dry matter)	8.59	8.49	8.55	8.39	8.25	8.33	8.42	8.61	8.44	8.50	8.47

Abbreviations: PBMP = poultry by-product meal protein, CCMP = conventional cottonseed meal, LGCMP = low-gossypol cotton seed meal. ^1^ Yongsheng Feed Corporation, Binzhou, China; proximate composition moisture, 7.28; CP 73.3; CL, 9.58. ^2^ American Proteins Inc., USA; proximate composition: moisture:4.38; CP: 68.67; CL: 14.74. ^3^ Chinatex Corporation, Xingjiang, China; CCM proximate composition: moisture, 6.88; CP 65.45; CL, 2.0. ^4^ Chinatex Corporation, Xingjiang, China; LGCM proximate composition: moisture, 8.09; CP 66.33, CL, 2.03.

**Table 2:** Amino acid compositions (dry matter basis, %) of experimental diets.

Ingredients	Dietary Treatments										
	PBMP	CCMP 20	CCMP 40	CCMP 60	CCMP 80	CCMP 100	LGCMP 20	LGCMP 40	LGCMP 60	LGCMP 80	LGCMP 100
Essential amino acids											
Lysine	3.32	3.29	3.28	3.26	3.24	3.23	3.21	3.25	3.26	3.28	3.29
Arginine	2.86	2.65	2.70	2.74	2.79	2.83	2.75	2.73	2.74	2.82	2.88
Methionine	1.69	1.81	1.85	1.91	1.93	1.90	1.76	1.82	1.85	1.93	1.92
Histidine	1.83	2.12	2.09	2.07	2.04	2.01	2.22	2.09	2.01	2.05	2.01
Leucine	1.96	2.05	2.13	2.21	2.29	2.38	2.05	2.13	2.21	2.29	2.38
Isoleucine	3.28	3.57	3.61	3.66	3.71	3.76	3.49	3.55	3.68	3.74	3.78
Valine	1.93	2.09	2.21	2.32	2.43	2.54	2.15	2.20	2.31	2.36	2.44
Phenylalanine	2.18	2.30	2.34	2.38	2.41	2.45	2.32	2.34	2.35	2.45	2.46
Threonine	1.41	1.41	1.43	1.46	1.48	1.51	1.40	1.44	1.45	1.46	1.52
Non-essential amino acids											
Glutamic acid	4.06	4.39	4.60	4.81	5.02	5.23	4.35	4.60	4.81	5.02	5.23
Serine	1.82	2.22	2.25	2.28	2.31	2.34	2.21	2.25	2.28	2.31	2.34
Aspartic acid	5.75	6.79	7.08	7.38	7.67	7.96	6.59	7.08	7.38	7.67	7.96
Tyrosine	2.89	3.87	3.52	3.17	2.83	2.48	3.89	3.58	3.19	2.93	2.49
Alanine	3.51	3.22	2.99	2.76	2.54	2.31	3.25	2.95	2.79	2.59	2.41
Cystine	0.42	0.40	0.36	0.31	0.26	0.22	0.41	0.36	0.31	0.26	0.22
Glycine	1.36	1.53	1.60	1.68	1.76	1.84	1.45	1.65	1.69	1.78	1.86
Proline	2.18	2.84	2.75	2.66	2.57	2.48	2.75	2.78	2.65	2.56	2.47

Abbreviations: PBMP = poultry by-product meal protein, CCMP = conventional cottonseed meal protein, LGCMP = low-gossypol cotton seed meal protein.

**Table 3:** Growth performance and feed utilization of hybrid grouper juveniles fed different experimental diets for 9 weeks.

Dietary Treatments	Type of Cottonseed Meal Proteome	Levels of Replacement	WG%	FCR	DFI	PER	Survival
PBMP		0	1068 ± 17 ^b^	0.91 ± 0.00 ^cd^	1.45 ± 0.01 ^cd^	2.38 ± 0.01 ^bc^	100
CCMP 20	CCMP	20	876 ± 9 ^d^	0.91 ± 0.00 ^cd^	1.45 ± 0.00 ^cd^	2.38 ± 0.01 ^bc^	100
CCMP 40	CCMP	40	907 ± 6 ^d^	0.92 ± 0.00 ^cd^	1.45 ± 0.00 ^cd^	2.37 ± 0.01 ^bc^	100
CCMP 60	CCMP	60	1000 ± 19 ^c^	0.91 ± 0.00 ^cd^	1.45 ± 0.00 ^cd^	2.38 ± 0.00 ^bc^	100
CCMP 80	CCMP	80	1166 ± 7 ^a^	0.90 ± 0.00 ^cd^	1.42 ± 0.01 ^cd^	2.43 ± 0.01 ^ab^	100
CCMP 100	CCMP	100	809 ± 10 ^e^	1.04 ± 0.01 ^a^	1.65 ± 0.02 ^a^	2.10 ± 0.02 ^e^	100
LGCMP 20	LGCMP	20	902 ± 8 ^d^	0.93 ± 0.00 ^c^	1.47 ± 0.01 ^c^	2.35 ± 0.01 ^c^	100
LGCMP 40	LGCMP	40	1211 ± 15 ^a^	0.89 ± 0.00 ^d^	1.41 ± 0.01 ^d^	2.45 ± 0.01 ^a^	100
LGCMP 60	LGCMP	60	1017 ± 8 ^bc^	0.91 ± 0.00 ^cd^	1.43 ± 0.01 ^cd^	2.40 ± 0.01 ^abc^	100
LGCMP 80	LGCMP	80	908 ± 16 ^d^	0.98 ± 0.01 ^b^	1.57 ± 0.01 ^b^	2.21 ± 0.01 ^d^	100
LGCMP 100	LGCMP	100	758 ± 14 ^e^	1.02 ± 0.02 ^a^	1.62 ± 0.02 ^a^	2.13 ± 0.03 ^e^	100
Means of main effects ^1^							100
	CCMP		951	0.93 ^B^	2.97 ^B^	2.33 ^A^	
	LGCMP		959	0.94 ^A^	3.00 ^A^	2.30 ^B^	
		20	889 ^c^	0.92 ^c^	2.91 ^c^	2.37 ^b^	
		40	1059 ^a^	0.90 ^c^	2.87 ^c^	2.41 ^a^	
		60	1009 ^b^	0.91 ^c^	2.89 ^c^	2.39 ^ab^	
		80	1037 ^ab^	0.94 ^b^	2.98 ^b^	2.32 ^c^	
		100	783 ^d^	1.03 ^a^	3.27 ^a^	2.11 ^d^	
Analysis of variance (*p*-value)							
Type of cottonseed meal proteome			0.321	0.023	0.017	0.006	
Levels of replacement			0.000	0.000	0.000	0.000	
Interaction			0.000	0.000	0.000	0.000	

Abbreviations: PBMP = poultry by-product meal protein, CCMP = conventional cottonseed meal, LGCMP = low-gossypol cotton seed meal, WG% = weight-gain percentage, FCR = feed conversion ratio, DFI = daily feed intake, PER = protein efficiency ratio. The values are expressed as mean ± variance, with different letter superscripts in the same column indicating significant differences (*p* < 0.05). ^1^ Main effect means followed by the different letter (CSM sources= upper case; PBMP replacement levels by CCMP or LGCMP = lower case).

**Table 4:** Body condition indices of hybrid grouper juveniles fed different experimental diets for 9 weeks.

Dietary Treatments	Type of Cottonseed Meal Proteome	Levels of Replacement	HSI	CF	IPF
PBMP		0	2.10 ± 0.10	1.98 ± 0.04	2.11 ± 0.07
CCMP 20	CCMP	20	2.19 ± 0.09	1.91 ± 0.05	1.96 ± 0.14
CCMP 40	CCMP	40	2.25 ± 0.10	2.04 ± 0.06	1.85 ± 0.15
CCMP 60	CCMP	60	2.33 ± 0.18	2.05 ± 0.04	1.76 ± 0.16
CCMP 80	CCMP	80	2.44 ± 0.11	2.14 ± 0.05	1.86 ± 0.11
CCMP 100	CCMP	100	1.98 ± 0.12	1.99 ± 0.03	1.74 ± 0.15
LGCMP 20	LGCMP	20	1.86 ± 0.19	2.02 ± 0.04	1.66 ± 0.09
LGCMP 40	LGCMP	40	1.94 ± 0.15	2.00 ± 0.04	1.55 ± 0.16
LGCMP 60	LGCMP	60	2.16 ± 0.12	2.10 ± 0.06	1.49 ± 0.10
LGCMP 80	LGCMP	80	2.22 ± 0.13	2.10 ± 0.04	1.59 ± 0.11
LGCMP 100	LGCMP	100	2.40 ± 0.06	2.12 ± 0.03	1.70 ± 0.14
Means of main effects ^1^					
	CCMP		2.24	2.24	1.84 ^A^
	LGCMP		2.12	2.12	1.60 ^B^
		20	2.03	2.03	1.81
		40	2.09	2.09	1.70
		60	2.25	2.25	1.63
		80	2.33	2.33	1.73
		100	2.19	2.19	1.72
Analysis of variance (*p*-value)					
Type of cottonseed meal proteome			0.074	0.131	0.010
Levels of replacement			0.228	0.132	0.738
Interaction			0.446	0.037	0.827

Abbreviations: PBMP = poultry by-product meal protein, CCMP = conventional cottonseed meal, LGCMP = low-gossypol cotton seed meal, CF = condition factor, HSI = hepatosomatic index, IPF = intraperitoneal fat. The values are expressed as mean ± variance, with different letter superscripts in the same column indicating significant differences (*p* < 0.05). ^1^ Main effect means followed by the different letter (CSM sources= upper case; PBMP replacement levels by CCMP or LGCMP = lower case) within the same row mean significantly different at *p* < 0.05.

**Table 5:** Whole-body and white muscle compositions of hybrid grouper juveniles fed different experimental diets for 9 weeks.

Dietary Treatments	Type of Cottonseed Meal Proteome	Levels of Replacement	Whole-Body Composition			White Muscle Composition		
			Moisture	Protein	Lipid	Moisture	Protein	Lipid
PBMP		0	70.1 ± 0.1	17.8 ± 0.0	7.44 ± 0.06 ^a^	76.8 ± 0.0	19.6 ± 0.3 ^c^	1.61 ± 0.19
CCMP 20	CCMP	20	70.6 ± 0.1	17.8 ± 0.1	7.13 ± 0.02 ^b^	76.2 ± 0.1	19.8 ± 0.1 ^bc^	1.42 ± 0.07
CCMP 40	CCMP	40	70.4 ± 0.1	17.7 ± 0.1	6.85 ± 0.02 ^c^	76.6 ± 0.1	19.8 ± 0.2 ^abc^	1.34 ± 0.09
CCMP 60	CCMP	60	70.7 ± 0.1	18.0 ± 0.1	6.56 ± 0.02 ^d^	76.5 ± 0.1	19.9 ± 0.2 ^abc^	1.25 ± 0.10
CCMP 80	CCMP	80	71.2 ± 0.0	18.2 ± 0.1	6.28 ± 0.01 ^e^	77.3 ± 0.1	20.0 ± 0.0 ^bc^	1.32 ± 0.01
CCMP 100	CCMP	100	71.5 ± 0.1	17.9 ± 0.3	6.09 ± 0.03 ^f^	77.2 ± 0.3	19.7 ± 0.2 ^abc^	1.00 ± 0.08
LGCMP 20	LGCMP	20	70.5 ± 0.2	18.1 ± 0.2	7.03 ± 0.03 ^bc^	76.0 ± 0.2	20.2 ± 0.2 ^ab^	1.40 ± 0.30
LGCMP 40	LGCMP	40	70.6 ± 0.1	18.3 ± 0.1	6.85 ± 0.02 ^c^	76.5 ± 0.1	20.3 ± 0.1 ^a^	1.63 ± 0.18
LGCMP 60	LGCMP	60	70.6 ± 0.	18.3 ± 0.1	6.58 ± 0.02 ^d^	76. 9 ± 0.1	20.0 ± 0.2 ^abc^	1.27 ± 0.18
LGCMP 80	LGCMP	80	70.4 ± 0.1	18.2 ± 0.1	6.33 ± 0.02 ^e^	76.6 ± 0.8	20.0 ± 0.1 ^abc^	1.21 ± 0.22
LGCMP 100	LGCMP	100	71.1 ± 0.1	18.1 ± 0.1		76.9 ± 0.1	19.9 ± 0.2 ^abc^	1.03 ± 0.10
Means of main effects ^1^								
	CCMP		70.87	17.93 ^B^	6.58	76.91 ^A^	19.85 ^B^	1.27
	LGCMP		70.77	18.12 ^A^	6.59	76.66 ^B^	20.08 ^A^	1.31
		20	70.35 ^c^	17.97	7.08 ^a^	76.10 ^d^	20.00	1.41 ^a^
		40	70.62 ^bc^	18.01	6.85 ^b^	76.62 ^c^	20.07	1.49 ^a^
		60	70.75 ^bc^	17.96	6.57 ^bc^	76.83 ^bc^	19.95	1.26 ^ab^
		80	70.89 ^b^	18.20	6.31 ^c^	77.05 ^ab^	19.87	1.26 ^ab^
		100	71.48 ^a^	18.00	6.15 ^d^	77.34 ^a^	19.95	1.02 ^b^
Analysis of variance (*p*-value)								
Type of cottonseed meal proteome			0.242	0.030	0.548	0.002	0.000	0.491
Levels of replacement			0.000	0.383	0.000	0.000	0.215	0.000
Interaction			0.051	0.295	0.148	0.752	0.019	0.276

Abbreviations: PBMP = poultry by-product meal protein, CCMP = conventional cottonseed meal, LGCMP = low-gossypol cotton seed meal. The values are expressed as mean ± variance, with different letter superscripts in the same column indicating significant differences (*p* < 0.05). ^1^ Main effect means followed by the different letter (CSM sources= upper case; PBMP replacement levels by CCMP or LGCMP = lower case). Main effect means followed by the different letter (CSM sources = upper case; rep= lower case) within the same row mean significantly different at *p* < 0.05.

**Table 6:** Gut histology of hybrid grouper juveniles fed different experimental diets for 9 weeks.

Dietary Treatments	Type of Cottonseed Meal Proteome	Levels of Replacement	Foregut (μm)		Midgut (μm)		Hindgut (μm)	
			hF	hMV	hF	hMV	hF	hMV
PBMP		0	448 ± 5 ^c^	3.0 ± 0.1 ^b^	385 ± 9 ^def^	2.5 ± 0.1 ^c^	423 ± 5 ^ef^	2.7 ± 0.1 ^fg^
CCMP 20	CCMP	20	451 ± 11 ^c^	2.9 ± 0.1 ^bcd^	387 ± 5 ^def^	2.6 ± 0.1 ^bc^	433 ± 5 ^cdef^	2.7 ± 0.1 ^efg^
CCMP 40	CCMP	40	465 ± 5 ^bc^	2.7 ± 0.0 ^d^	408 ± 3 ^bcd^	2.6 ± 0.1 ^bc^	439 ± 7 ^bcde^	2.8 ± 0.1 ^cdef^
CCMP 60	CCMP	60	470 ± 5 ^bc^	2.9 ± 0.0 ^bcd^	439 ± 14 ^a^	2.8 ± 0.1 ^ab^	459 ± 3 ^ab^	2.9 ± 0.0 ^bc^
CCMP 80	CCMP	80	517 ± 9 ^a^	3.2 ± 0.1 ^a^	427 ± 6 ^ab^	2.8 ± 0.1 ^ab^	473 ± 7 ^a^	3.3 ± 0.0 ^a^
CCMP 100	CCMP	100	456 ± 11 ^bc^	2.8 ± 0.0 ^cd^	395 ± 7 ^de^	2.7 ± 0.1 ^abc^	445 ± 7 ^bcd^	2.9 ± 0.0 ^cde^
LGCMP 20	LGCMP	20	460 ± 7 ^bc^	3.0 ± 0.1 ^bc^	366 ± 9 ^f^	2.6 ± 0.0 ^bc^	414 ± 11 ^f^	2.6 ± 0.0 ^g^
LGCMP 40	LGCMP	40	513 ± 7 ^a^	3.3 ± 0.1 ^a^	424 ± 12 ^abc^	2.9 ± 0.1 ^a^	476 ± 12 ^a^	3.0 ± 0.1 ^ab^
LGCMP 60	LGCMP	60	478 ± 8 ^b^	3.0 ± 0.1 ^bc^	401 ± 6 ^cde^	2.8 ± 0.1 ^ab^	449 ± 2 ^bc^	2.9 ± 0.1 ^cd^
LGCMP 80	LGCMP	80	466 ± 4 ^bc^	2.9 ± 0.0 ^bc^	382 ± 11 ^ef^	2.7 ± 0.0 ^abc^	433 ± 6 ^cdef^	2.7 ± 0.0 ^defg^
LGCMP 100	LGCMP	100	459 ± 15 ^bc^	2.8 ± 0.1 ^bcd^	369 ± 6 ^f^	2.6 ± 0.0 ^bc^	425 ± 9 ^def^	2.5 ± 0.1 ^h^
Means of main effects ^1^								
	CCMP		472.32	2.90 ^B^	411.49 ^A^	2.69	450.38 ^A^	2.93 ^A^
	LGCMP		475.61	3.00 ^A^	388.78 ^B^	2.69	439.78 ^B^	2.79 ^B^
		20	456.13 ^c^	2.91 ^bc^	376.63 ^c^	2.58 ^b^	423.99 ^b^	2.70 ^b^
		40	489.41 ^ab^	3.02 ^ab^	416.62 ^ab^	2.73 ^a^	458.11 ^a^	2.96 ^a^
		60	474.21 ^b^	2.93 ^bc^	420.43 ^a^	2.76 ^a^	454.46 ^a^	2.93 ^a^
		80	492.03 ^a^	3.08 ^a^	404.85 ^b^	2.72 ^a^	453.26 ^a^	3.00 ^a^
		100	458.05 ^c^	2.81 ^c^	382.17 ^c^	2.66 ^ab^	435.60 ^b^	2.70 ^b^
Analysis of variance (*p*-value)								
Type of cottonseed meal proteome			0.327	0.001	0.000	0.768	0.001	0.000
Levels of replacement			0.000	0.000	0.000	0.004	0.000	0.000
Interaction			0.000	0.000	0.000	0.002	0.000	0.000

Abbreviations: PBMP = poultry by-product meal protein, CCMP = conventional cottonseed meal, LGCMP = low-gossypol cotton seed meal, hF = fold height. hMV = microvillus height. The values are expressed as mean ± variance, with different letter superscripts in the same column indicating significant differences (*p* < 0.05). ^1^ Main effect means followed by the different letter (CSM sources= upper case; PBMP replacement levels by CCMP or LGCMP = lower case).

## Data Availability

Data is available from the corresponding author upon request.
